# In vivo silencing of amphiregulin by a novel effective Self-Assembled-Micelle inhibitory RNA ameliorates renal fibrosis via inhibition of EGFR signals

**DOI:** 10.1038/s41598-021-81726-2

**Published:** 2021-01-26

**Authors:** Seung Seob Son, Soohyun Hwang, Jun Hong Park, Youngho Ko, Sung-Il Yun, Ji-Hye Lee, Beomseok Son, Tae Rim Kim, Han-Oh Park, Eun Young Lee

**Affiliations:** 1 siRNAgen Therapeutics, Daejeon, 34302 Republic of Korea; 2 Bioneer Corporation, 8-11 Munpyeongseo-ro, Daedeok-gu, Daejeon, 34302 Republic of Korea; 3 Department of Pathology, Soonchunhyang University Cheonan Hospital, Cheonan, 31151 Republic of Korea; 4 Department of Internal Medicine, Soonchunhyang University Cheonan Hospital, 31 Soonchunhyang 6-gil, Cheonan, 31151 Republic of Korea; 5 Institute of Tissue Regeneration, College of Medicine, Soonchunhyang University, Cheonan, 31151 Republic of Korea; 6 BK21 FOUR Project, College of Medicine, Soonchunhyang University, Cheonan, 31151 Republic of Korea

**Keywords:** Chronic kidney disease, Renal fibrosis

## Abstract

Amphiregulin (AREG) is a transmembrane glycoprotein recently implicated in kidney fibrosis. Previously, we reported that the AREG-targeting Self-Assembled-Micelle inhibitory RNA (SAMiRNA-AREG) alleviated fibrosis by stably silencing the *AREG* gene, and reduced the side effects of conventional siRNA treatment of pulmonary fibrosis. However, the therapeutic effect of SAMiRNA-AREG in renal fibrosis has not been studied until now. We used two animal models of renal fibrosis generated by a unilateral ureteral obstruction (UUO) and an adenine diet (AD) to investigate whether SAMiRNA-AREG inhibited renal fibrosis. To investigate the delivery of SAMiRNA-AREG to the kidney, Cy5-labeled SAMiRNA-AREG was injected into UUO- and AD-induced renal fibrosis models. In both kidney disease models, SAMiRNA-AREG was delivered primarily to the damaged kidney. We also confirmed the protective effect of SAMiRNA-AREG in renal fibrosis models. SAMiRNA-AREG markedly decreased the UUO- and AD-induced AREG mRNA expression. Furthermore, the mRNA expression of fibrosis markers, including α-smooth muscle actin, fibronectin, α1(I) collagen, and α1(III) collagen in the UUO and AD-induced kidneys, was diminished in the SAMiRNA-AREG-treated mice. The transcription of inflammatory markers (tumor necrosis factor-α and monocyte chemoattractant protein-1) and adhesion markers (vascular cell adhesion molecule 1 and intercellular adhesion molecule 1) was attenuated. The hematoxylin and eosin, Masson’s trichrome, and immunohistochemical staining results showed that SAMiRNA-AREG decreased renal fibrosis, AREG expression, and epidermal growth factor receptor (EGFR) phosphorylation in the UUO- and AD-induced models. Moreover, we studied the effects of SAMiRNA-AREG in response to TGF-β1 in mouse and human proximal tubule cells, and mouse fibroblasts. TGF-β1-induced extracellular matrix production and myofibroblast differentiation were attenuated by SAMiRNA-AREG. Finally, we confirmed that upregulated AREG in the UUO or AD models was mainly localized in the distal tubules. In conclusion, SAMiRNA-AREG represents a novel siRNA therapeutic for renal fibrosis by suppressing EGFR signals.

## Introduction

The prevalence of chronic kidney disease (CKD) is increasing worldwide. It is also increasing in Korea. However, no effective therapeutic drugs are available for the disease^1,2^. Renal interstitial fibrosis results in CKD. The differentiation of fibroblasts into myofibroblasts in the renal interstitial space is the major cause of fibrosis^3–5^. The unilateral ureteral obstruction (UUO) model exhibits the symptoms of severe CKD patients. The UUO model is characterized by damaged tubular morphology, the infiltration of macrophages, interstitial inflammation, and the elevated expression of cytokines and myofibroblast markers such as α-smooth muscle actin (α-SMA)^6–9^. Previous studies have shown that an adenine diet (AD) induced CKD via tubulointerstitial nephropathy^10^. Adenine converted to 2,8-dihydroxyadenine accumulates in the urine and renal tubules, inducing renal damage^10^. As a result, remarkable histological changes including interstitial fibrosis and crystal deposition occur in the renal proximal tubules. In this model, the mRNA expression of fibrosis markers was increased by tubular fibrosis.

Amphiregulin (AREG), which is the ligand for epidermal growth factor receptor (EGFR), plays an important role in wound healing, as well as various aspects of tumorigenesis, metastasis, and angiogenesis in cancer tissue^11,12^. AREG is known as a growth factor involved in the differentiation of fibroblasts to myofibroblasts and their proliferation. It is also an autocrine growth factor as well as a T-cell regulator^13^. Recently, AREG has been identified as an important factor for myofibroblast proliferation in many diseases, and AREG combined with EGFR amplified renal fibrosis in the proximal tubules^14–17^. AREG was also detected in the urine of patients with CKD and acute kidney injury^18^.

Small interfering RNA (siRNA) is a class of double-stranded non-coding RNA widely used to regulate gene expression in vitro and in vivo. As a disease-modifying agent, siRNA stands out as a promising therapeutic platform that can be designed to modulate gene expression patterns and treat currently incurable diseases^19,20^. However, the effectiveness of in vivo delivery beyond the liver and nonspecific innate immune stimulation by siRNA are factors limiting the therapeutic development of siRNAs^19,21,22^. To overcome these limitations, we developed Self-Assembled-Micelle inhibitory RNA (SAMiRNA). These DNA/RNA hybrids made of individual bi-conjugated siRNA with a hydrophilic polymer and hydrocarbon tail on each end spontaneously formed stable nanoparticles in solutions. SAMiRNA dramatically enhanced RNA stability in physiological conditions and in vivo silencing efficacy^23^. In particular, SAMiRNA designed to knockdown AREG (SAMiRNA-AREG), showed minimal innate immunostimulatory effects compared to unmodified siRNAs or other stable nucleic acid–lipid particle liposome complexes^23^. SAMiRNA-AREG is integrated with the RNA-induced silencing complex (RISC) after endocytosis by the cleavage of hydrophobic conjugate and subsequent binding to Ago2 in the cytoplasm. We confirmed the level of antisense RNA in RISC by Ago2 immunoprecipitation and real-time quantitative reverse transcription PCR (qRT-PCR) analysis (data not shown). The size of the SAMiRNA-AREG nanoparticles varied from 10 to 100 nm and carried a neutral charge, which is ideal for delivery and reduced toxicity. SAMiRNA-AREG was stable in the bloodstream and facilitated the delivery of unmodified oligonucleotides to the target cells.

SAMiRNA-AREG represents a novel therapeutic strategy against many diseases. Previously, we demonstrated AREG targeting in a lung fibrosis model via intravenous (i.v.) injection of SAMiRNA-AREG nanoparticles^23^. SAMiRNA-AREG was developed and tested in pulmonary disease models, such as bleomycin-induced pulmonary fibrosis and transforming growth factor-beta (TGF-β) transgenic mouse models^23^. The SAMiRNA-AREG platform has not only been successfully used for drug delivery to the lungs but also showed no hepatotoxicity^23^. In damaged vasculature or fibrosis, SAMiRNA-AREG can be selectively delivered to inflamed or fibrotic tissues by the enhanced permeation and retention (EPR) effect^24,25^.

In this study, we reported the efficient delivery of SAMiRNA-AREG into kidney lesions. These findings suggest that SAMiRNA-AREG nanoparticles can be used as an effective siRNA therapeutic modality in renal fibrosis. We also demonstrated the anti-fibrotic and anti-inflammatory effects of SAMiRNA-AREG using two different mouse models of renal fibrosis induced by either UUO or AD. The anti-fibrotic effects of SAMiRNA-AREG were confirmed in mouse and human proximal tubule cells and mouse fibroblasts stimulated by TGF-β. Taken together, our results indicated that SAMiRNA-AREG has therapeutic effects on renal fibrosis.

## Results

### AREG is increased in renal fibrosis

**Figure 1:**
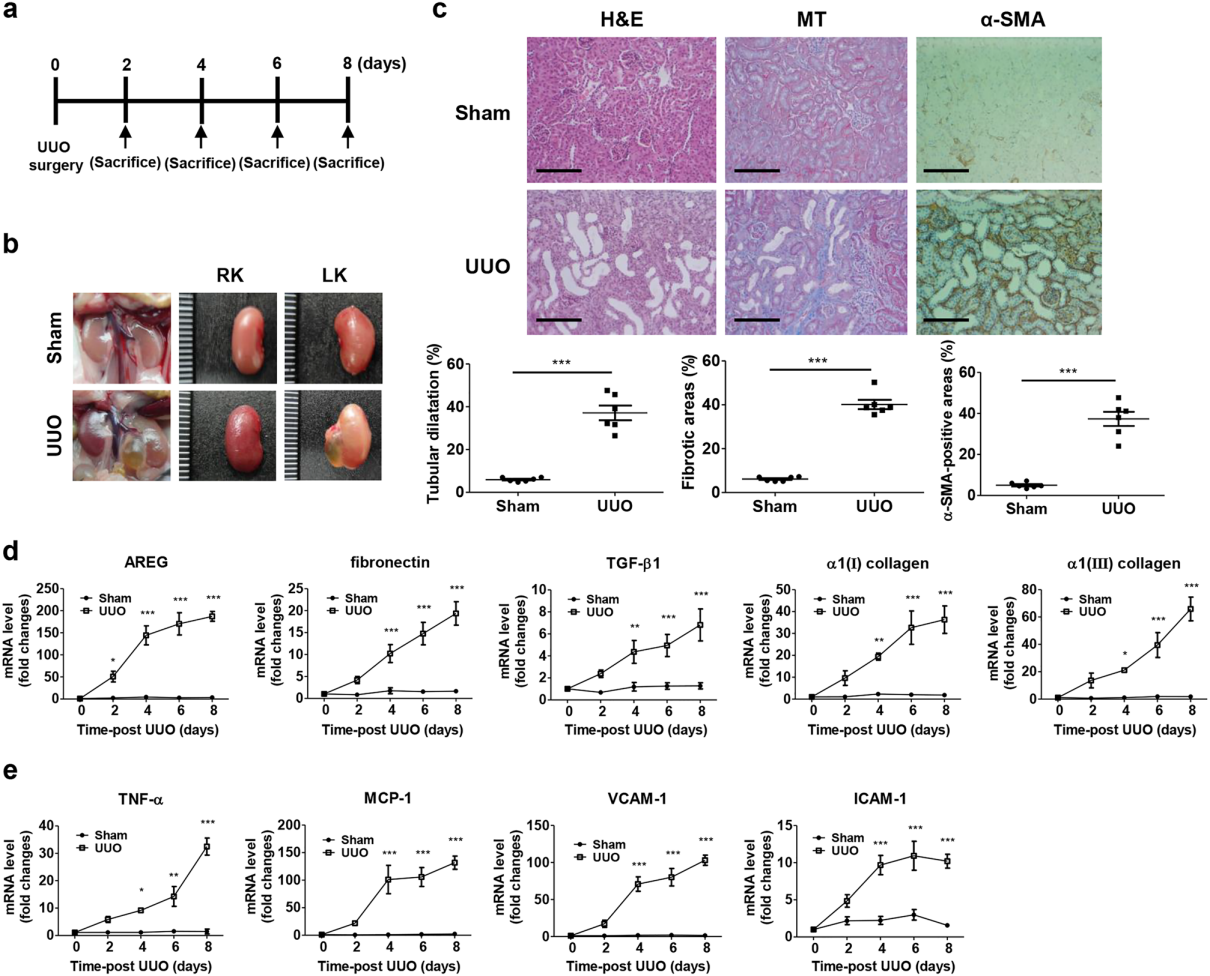
Establishment of UUO-induced mice and kinetic analysis of AREG expression, fibrosis-related genes, and inflammatory genes in the kidneys. (**a**) Time scheme for UUO-induced mice verification. (**b**) Representative images of kidneys from control or UUO-induced mice (harvested eight days after UUO surgery). (**c**) Histological analysis by H&E, MT, and IHC staining of control and UUO mice. Tubular dilatation, the fibrotic areas, and the α-SMA-positive-areas were quantified. Scale bar, 100 μm. (**d**) The kinetics of the expression of AREG, fibronectin, TGF-β1, α1(I) collagen, and α1(III) collagen in the kidneys of UUO mice. (**e**) Kinetics of the expression of inflammatory genes (TNF-α and MCP-1) and molecular adhesion genes (VCAM-1 and ICAM-1) in UUO-induced kidneys were measured using real-time qRT-PCR. The values in each panel represent the mean ± SEM of six mice induced by UUO. *p < 0.05, **p < 0.01, ***p < 0.001 compared to sham mice.

To establish CKD mouse models, C57BL/6 mice underwent UUO surgery, which is a well-known regimen inducing CKD (Fig. 1a). Eight days after UUO surgery, the structure of the kidneys was distinct from the control kidneys (Fig. 1b). Hematoxylin and eosin (H&E) and Masson’s trichrome (MT) staining showed that UUO surgery induced significant collagen deposition and fibrosis in the kidneys (Fig. 1c). We also detected elevated levels of α-SMA in the UUO kidneys through immunohistochemical (IHC) staining (Fig. 1c). The body weight, blood urea nitrogen (BUN), and serum creatinine levels were not significantly altered in the kidneys of UUO mice compared to the control mice (Supplementary Table S1), as previously reported^26^.

To identify the changes in AREG, fibrotic genes, inflammatory genes, and adhesion molecules, the mRNA expression levels of the UUO kidneys were analyzed and compared with the contralateral kidneys. The levels of AREG mRNA expression were significantly higher on day 2 in the UUO kidneys compared to the sham kidneys, and also dramatically increased on day 4 in the UUO kidneys. The expression of fibrotic genes, namely fibronectin, α1(I) collagen, α1(III) collagen, and TGF-β1, was significantly increased in the UUO kidneys compared to the sham kidneys (Fig. 1d). In particular, AREG increased at the early stages compared to the fibrosis markers. Likewise, the expression of tumor necrosis factor-α (TNF-α), monocyte chemoattractant protein 1 (MCP-1), which are inflammatory genes, was significantly upregulated on day 4 in the UUO kidneys compared to the sham kidneys. We also investigated the mRNA expression of adhesion molecules, vascular cell adhesion molecule 1 (VCAM-1) and intercellular adhesion molecule 1 (ICAM-1), which was dramatically increased on day 4 in the UUO kidneys compared to the sham kidneys (Fig. 1e).

**Figure 2:**
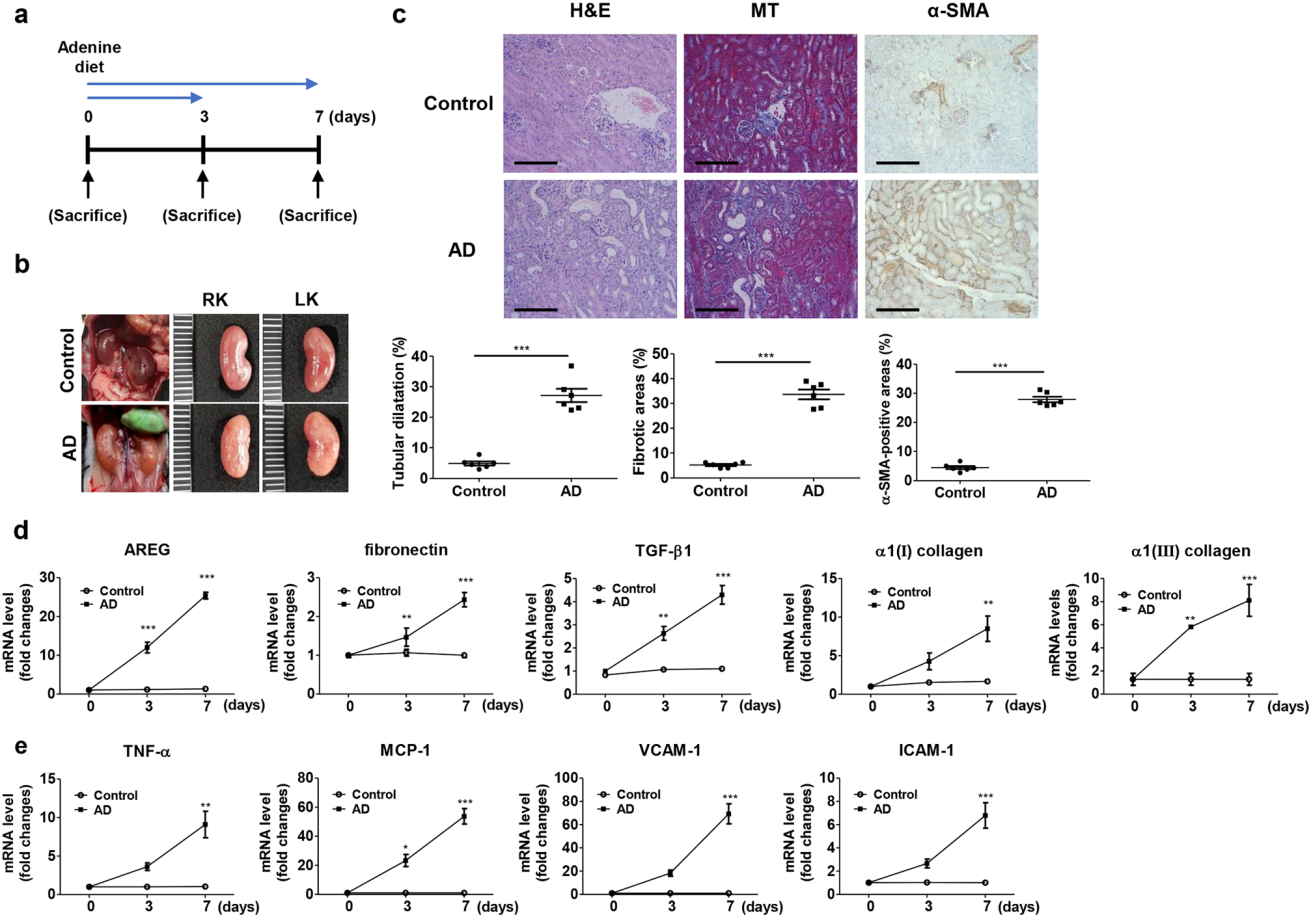
Establishment of AD-induced mice and kinetic analysis of AREG expression, fibrosis-related genes, and inflammatory genes in the kidneys. (**a**) Time scheme for AD-induced mice verification. (**b**) Representative images of kidneys from control or AD-induced mice (harvested seven days after AD). (**c**) Histological analysis by H&E, MT, and IHC staining of control and AD mice. Tubular dilatation, the fibrotic areas, and the α-SMA-positive areas were quantified. Scale bar, 100 μm. (**d**) Kinetics of the expression of AREG, fibronectin, TGF-β1, α1(I) collagen, and α1(III) collagen in the kidneys of AD mice. (**e**) The kinetics of the expression of inflammatory genes (TNF-α and MCP-1) and molecular adhesion genes (VCAM-1 and ICAM-1) in AD-induced kidneys were measured using real-time qRT-PCR. The values in each panel represent the mean ± SEM of six mice induced by AD. **p < 0.01, ***p < 0.001 compared to control mice.

Additionally, renal disease was induced by administering dietary adenine to C57BL/6 mice (Fig. 2a). The AD-induced model manifested CKD via tubulointerstitial nephropathy^27^. Swelling, discoloration, and deformation of renal tissues were observed in the mice treated with AD. Body weights were also altered by AD^10^ (Supplementary Table S1). After seven days of AD feeding, the structure of the kidneys changed significantly compared to the control kidneys (Fig. 2b). The BUN and serum creatinine levels were upregulated in kidneys after seven days of AD treatment compared to the control kidneys (Supplementary Table S1). H&E staining revealed severe morphological changes, such as tubular dilatation and atrophy, in the tubules of the AD-treated kidneys compared to the control kidneys (Fig. 2c). We also detected fibrotic lesions through MT staining. IHC staining showed increased levels of α-SMA in the kidneys treated with AD for seven days (Fig. 2c). To investigate the changes in the expression of the AREG gene and fibrotic genes, the mRNA expression of the AD model was analyzed and compared to that of the control kidneys. The level of AREG mRNA expression was significantly higher in the AD model on day 3 compared to that in the control mice and gradually increased by day 7 in AD-treated kidneys. The expression of TGF-β1 and the fibrotic genes such as fibronectin, α1(I) collagen, and α1(III) collagen, was significantly increased in AD-treated kidneys compared with control kidneys. It was increased in a time-dependent manner in AD-treated kidneys (Fig. 2d). In AD-treated kidneys, the AREG levels tended to increase in the early stages compared to the fibrotic markers. The mRNA expression of inflammatory markers and adhesion molecules was also elevated seven days after AD treatment (Fig. 2e).

### AREG inhibition by SAMiRNA-AREG prevents the development of renal fibrosis in the UUO model

**Figure 3:**
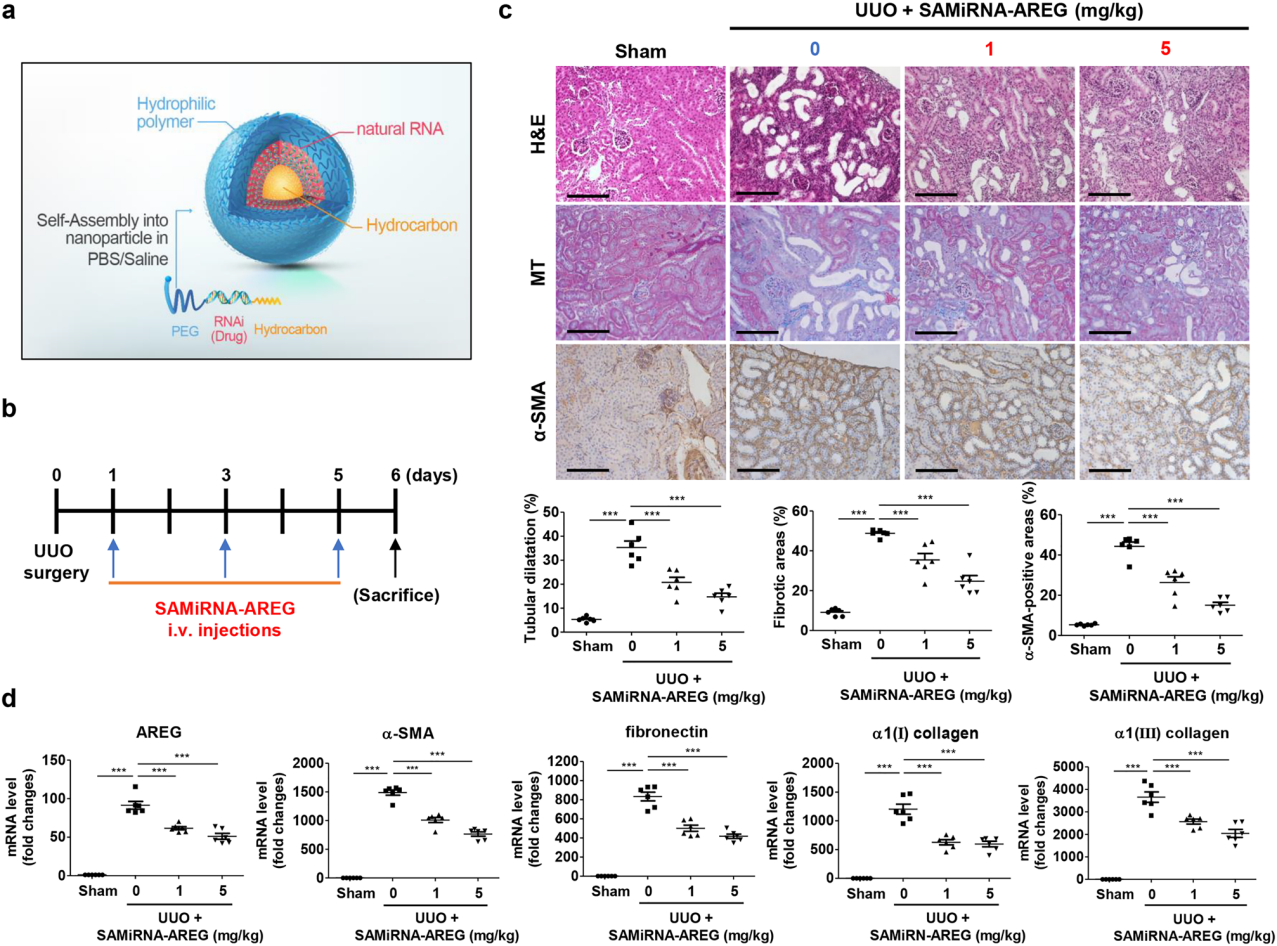
SAMiRNA-AREG ameliorated kidney fibrosis in the UUO-induced model. (**a**) The structure of SAMiRNA-AREG. (**b**) Time scheme for SAMiRNA-AREG efficacy analysis in UUO mice. (**c**) Histological analysis by H&E, MT, and IHC staining of control, UUO, and SAMiRNA-AREG-treated UUO mice. Tubular dilatation, the fibrotic areas, and the α-SMA-positive areas were quantified. Scale bar, 100 μm. (**d**) Kidney tissue lysates were subjected to real-time qRT-PCR analysis for AREG, α-SMA, fibronectin, α1(I) collagen, and α1(III) collagen. ***p < 0.001 compared with UUO mice by ANOVA with the Newman-Keuls post-hoc test.

As our previous study demonstrated that SAMiRNA-AREG, whose structure is shown in Fig. 3a inhibited the formation of pulmonary fibrosis in mouse models^23^, we analyzed the effects of SAMiRNA-AREG on UUO-induced kidney fibrosis. C57BL/6 mice were treated with SAMiRNA-AREG (5 mg/kg) three times via i.v. injections on days 1, 3, and 5 after UUO surgery (Fig. 3b). The H&E staining results revealed severe morphological changes in the tubules of the UUO-induced kidneys compared to the sham-operated kidneys (Fig. 3c). The changes included tubular dilatation, atrophy, interstitial fibrosis, and the infiltration of mononuclear cells. In contrast, the UUO-induced kidney injuries treated with SAMiRNA-AREG were ameliorated. We also performed MT staining to investigate the therapeutic role of SAMiRNA-AREG in renal fibrosis (Fig. 3c). The deposition of collagen in the interstitial sites was confirmed in the UUO-induced kidneys compared to the SAMiRNA-AREG-treated kidneys. We found that α-SMA was upregulated in myofibroblasts from the peritubular and the interstitial regions of UUO kidneys compared to those from mice treated with SAMiRNA-AREG (Fig. 3c). We investigated the effects of SAMiRNA-AREG on the expression of fibrotic markers. In UUO-induced kidneys, the mRNA expression of AREG and fibrotic markers increased, which was attenuated by SAMiRNA-AREG treatment (Fig. 3d).

### AREG inhibition by SAMiRNA-AREG prevents the development of renal fibrosis in the AD model

**Figure 4:**
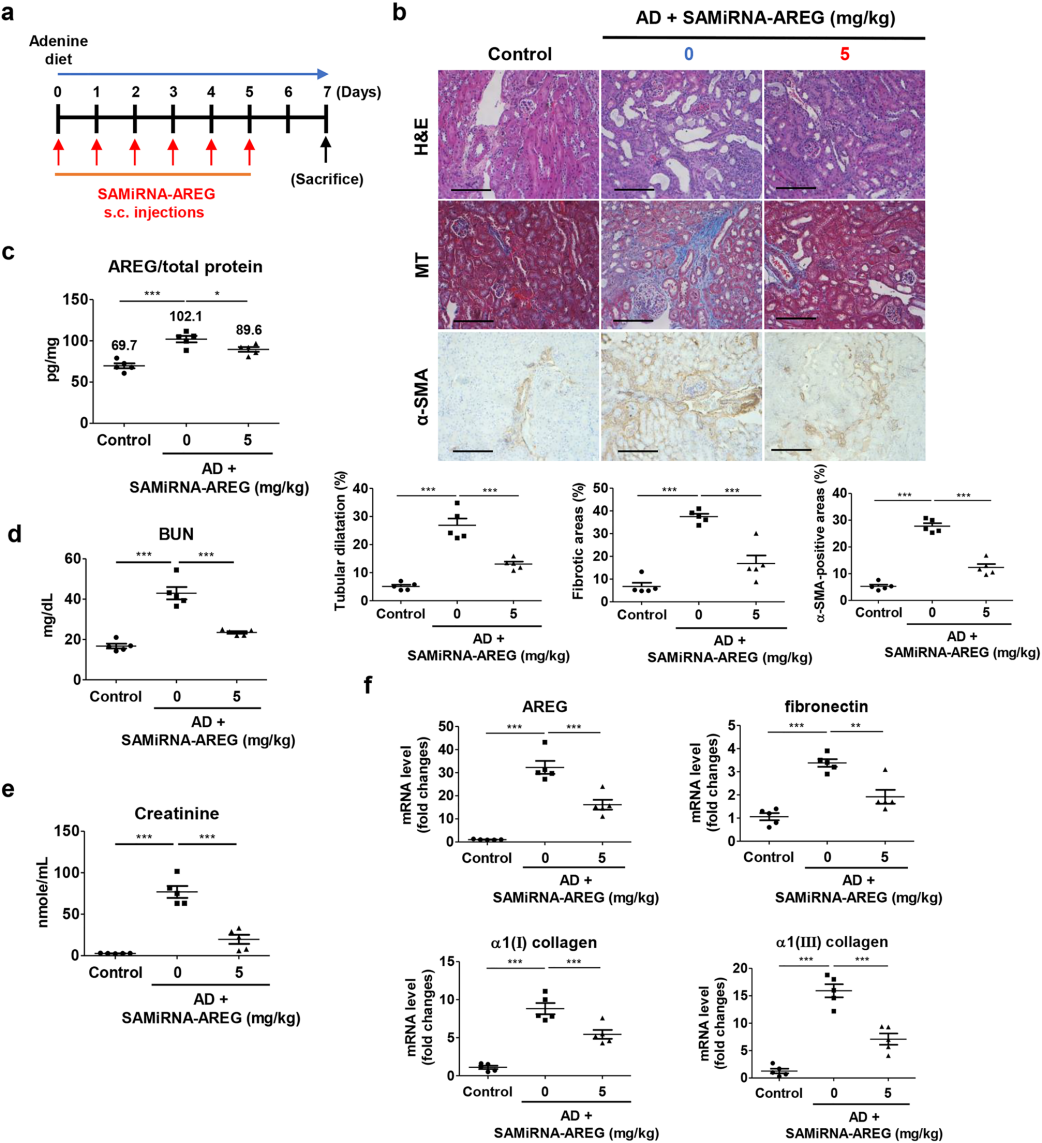
SAMiRNA-AREG ameliorated renal fibrosis in the AD-induced model. (**a**) Time scheme for SAMiRNA-AREG efficacy analysis in AD mice. (**b**) Histological analysis by H&E, MT, and IHC staining of control, UUO, or SAMiRNA-AREG-treated AD mice. Tubular dilatation, fibrosis areas, and α-SMA-positive areas were quantified. Scale bar, 100 μm. (**c**) AREG proteins in kidney tissue lysates were measured by ELISA. The numbers indicate the mean value of each data set. (**d**,**e**) BUN and serum creatinine were measured by ELISA. (**f**) Kidney tissue lysates were subjected to real-time qRT-PCR analysis for AREG, fibronectin, α1(I) collagen, and α1(III) collagen (mean ± SEM). *p < 0.05, **p < 0.01, ***p < 0.001 compared with AD mice by ANOVA with the Newman-Keuls post-hoc test.

We next analyzed the effect of SAMiRNA-AREG on AD-induced kidney fibrosis. Based on the kinetics of AREG and fibrotic marker expression shown in Fig. 2d, C57BL/6 mice were treated with SAMiRNA-AREG (5 mg/kg) daily via subcutaneous (s.c.) injections for 5 days (Fig. 4a). The H&E and MT staining results revealed severe morphological changes in the tubules of the AD-induced kidneys, including tubular dilatation and atrophy, and interstitial fibrosis, compared to the control kidneys (Fig. 4b). In contrast, SAMiRNA-AREG attenuated AD-induced morphological changes and fibrosis in the kidneys and reduced α-SMA expression (Fig. 4b). The AREG protein levels were downregulated in the AD-induced kidneys treated with SAMiRNA-AREG (Fig. 4c). We also evaluated the serum biochemical analysis data from the AD model and found that the BUN and the serum creatinine levels were increased in the AD model and decreased by SAMiRNA-AREG treatment (Fig. 4d,e). The mRNA expression of AREG and fibrosis markers was increased in AD-induced kidneys compared to that from mice treated with SAMiRNA-AREG (Fig. 4f).

### SAMiRNA-AREG specifically targets the kidney injury site

**Figure 5:**
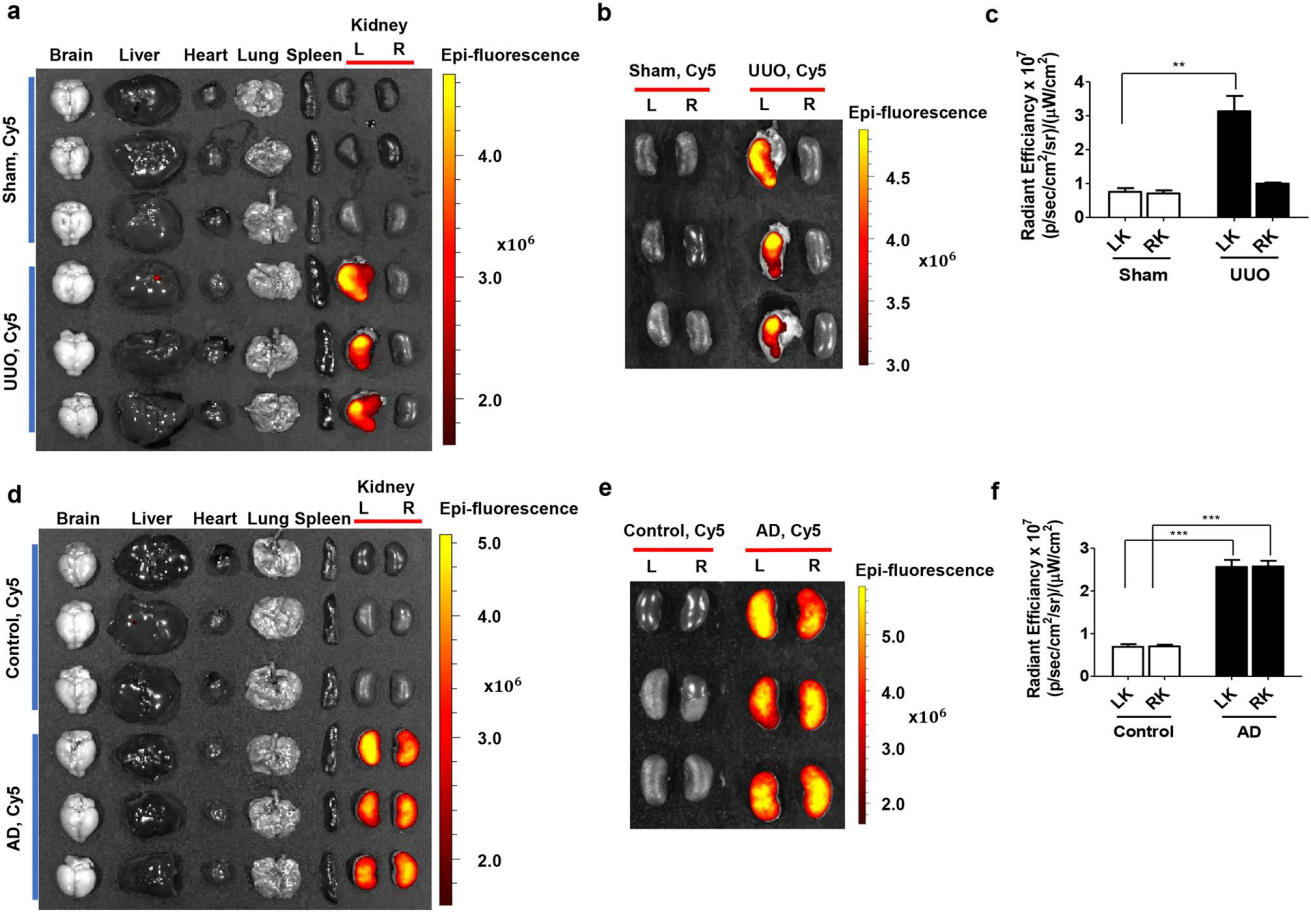
In vivo biodistribution of Cy5-labeled SAMiRNA-AREG in murine UUO- or AD-induced model of renal fibrosis. (**a**) Mice subjected to sham operations or 1 week after UUO were administered Cy5-labeled SAMiRNA-AREG via i.v. injection. Ex vivo fluorescent imaging was performed on organs including the brain, liver, heart, lung, spleen, and kidneys (L, obstructed, R, unobstructed) 48 h after the injection of Cy5-labeled SAMiRNA-AREG to sham or UUO mice, showing the Cy5 signal confined to the renal region. (**b**) Ex vivo fluorescent imaging of Cy5 signal from sham or UUO kidneys 48 h after the injection of the Cy5-labeled SAMiRNA-AREG. (**c**) Quantification of fluorescence intensity in the obstructed (LK) or unobstructed (RK) kidneys. (**d**) Mice fed a normal diet or 1 week of AD were subcutaneously injected with Cy5-labeled SAMiRNA-AREG. Ex vivo fluorescent imaging was performed on organs including the brain, liver, heart, lung, spleen, and kidneys 48 h after the injection of Cy5-labeled SAMiRNA-AREG to control or AD mice, showing the Cy5 signal confined to the renal region. (**e**) Ex vivo fluorescent imaging of Cy5 signal derived from control kidneys or AD model kidneys 48 h after the injection of Cy5-labeled SAMiRNA-AREG. (**f**) Quantification of fluorescence intensity of the kidneys in control and AD mice. The values in each panel represent the mean ± SEM of a minimum of three mice subjected to UUO or AD. **p < 0.01, ***p < 0.001 compared to UUO (LK) or AD (LK or RK) mice.

To investigate the delivery of SAMiRNA-AREG in renal disease, ex vivo fluorescence images were obtained 48 h after i.v. injections of Cy5-labeled SAMiRNA-AREG. A strong fluorescent signal was observed in the lesions of the UUO kidneys 48 h after the injection (Fig. 5a). The sham mouse model showed no obvious fluorescence kidney at the same time point. In contrast, the UUO-injured model showed a dramatic increase in the delivery efficiency of SAMiRNA-AREG (Fig. 5b). Intriguingly, the quantitative fluorescence levels remained high in the UUO kidneys after i.v. injections of SAMiRNA-AREG, but no significant increase in fluorescence levels was observed in the contralateral kidneys of the UUO mice (Fig. 5c). These findings suggest the enhanced accumulation of SAMiRNA-AREG in the diseased kidney sites after i.v. injection.

Additionally, to investigate the delivery of SAMiRNA-AREG to the injured kidneys compared to the control kidneys via s.c. injection, ex vivo fluorescence images were obtained 48 h after the injection of SAMiRNA-AREG labeled with Cy5. The ex vivo fluorescence was dramatically increased in the kidneys of AD mice (Fig. 5d). No significant delivery of SAMiRNA-AREG was found in the kidneys of the control mice, but the kidneys of AD-fed mice showed a dramatic increase in SAMiRNA-AREG delivery efficiency (Fig. 5e). The quantitative fluorescence levels in kidneys exposed to AD remained stronger than in the control kidneys (Fig. 5f). These findings suggest that SAMiRNA-AREG was also efficiently delivered to the diseased kidney sites via s.c. injection.

### AREG inhibition by SAMiRNA-AREG attenuates the expression of inflammatory cytokines and adhesion molecules in the renal fibrosis model

**Figure 6:**
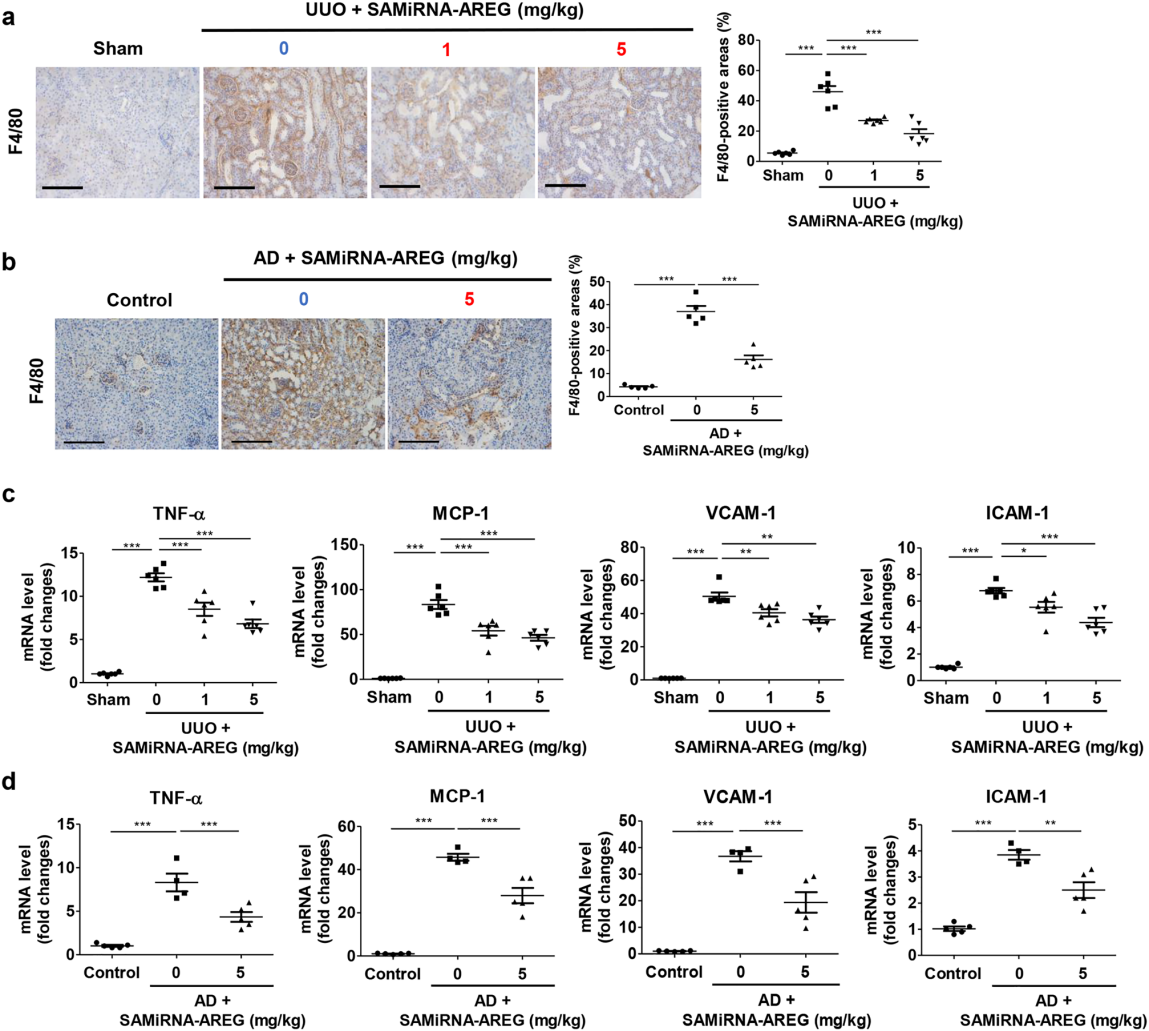
SAMiRNA-AREG ameliorated inflammatory cytokine levels and adhesion molecules in UUO- or AD-induced models. (**a**,**b**) Representative images of F4/80 expression in UUO and AD mice. The F4/80-positive areas were quantified. Scale bar, 100 μm. (**c**,**d**) Kidney tissue lysates were subjected to real-time qRT-PCR analysis of inflammatory markers (TNF-α and MCP-1) and adhesion molecules (VCAM-1 and ICAM-1) using RPL13A as the standard. *p < 0.05, **p < 0.01, ***p < 0.001 compared to UUO or AD mice.

To verify that elevated inflammation in the CKD mouse models was attenuated by SAMiRNA-AREG, inflammatory cytokine expression was evaluated in the kidneys from UUO-induced or AD-fed mice. The upregulation of F4/80, a macrophage subpopulation marker, in the UUO or AD kidneys was inhibited by SAMiRNA-AREG (Fig. 6a,b). Furthermore, the increases in TNF-α and MCP-1 mRNA expression in UUO- or AD-induced kidneys were reduced by SAMiRNA-AREG treatment (Fig. 6c,d). Upon kidney injury, adhesion molecules such as VCAM-1 and ICAM-1 were expressed to recruit inflammatory cells to the kidney injury site, followed by diffusion through vascular leaks. The mRNA expression of VCAM-1 and ICAM-1 was increased in UUO- or AD-treated kidneys, but SAMiRNA-AREG treatment significantly reduced their expression (Fig. 6c,d).

### SAMiRNA-AREG inhibits AREG expression and EGFR phosphorylation in renal fibrosis models

**Figure 7:**
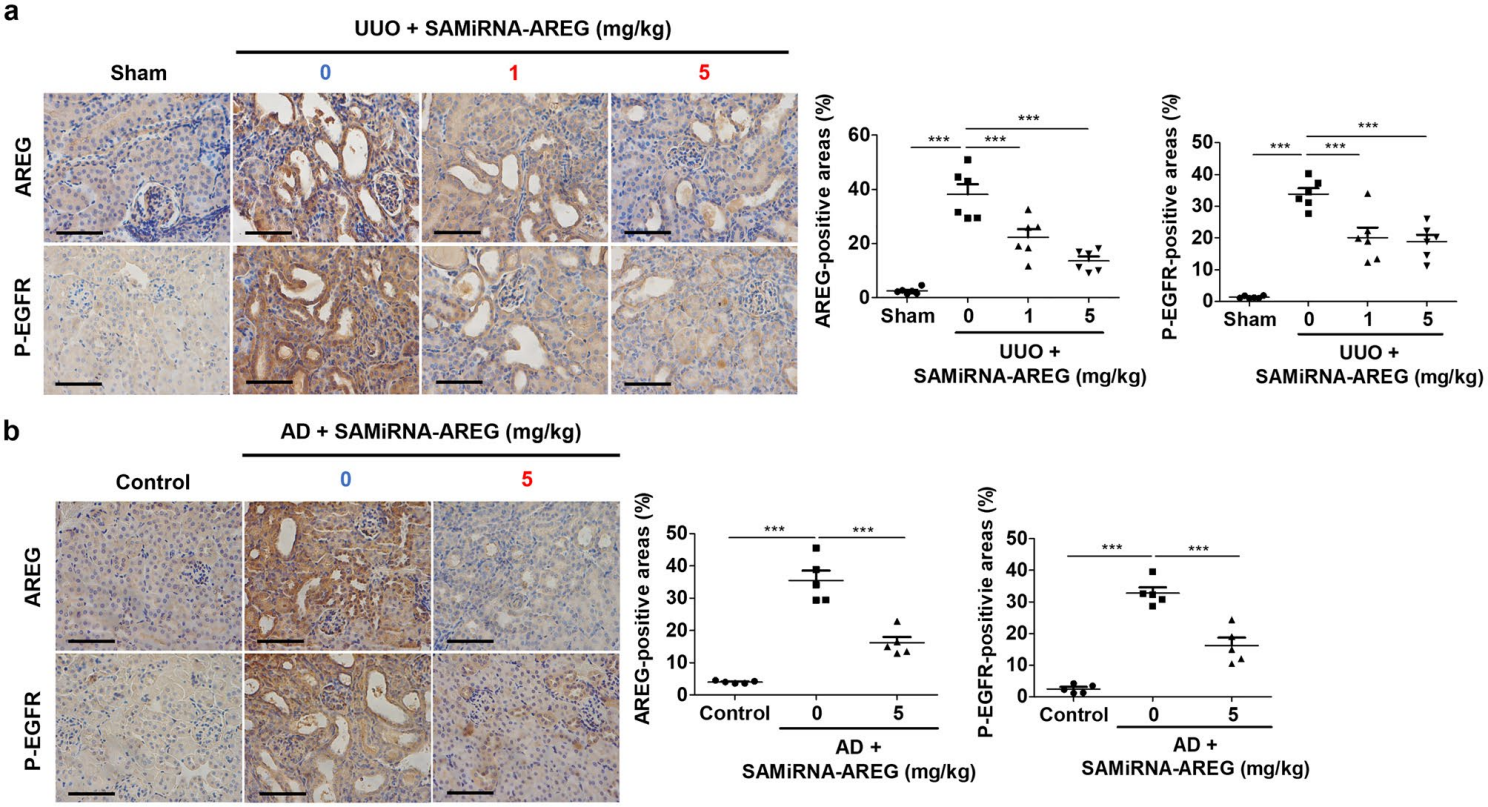
SAMiRNA-AREG inhibited EGFR phosphorylation by downregulating AREG in UUO- or AD-induced renal fibrosis. (**a**,**b**) Representative images of AREG expression and EGFR phosphorylation revealed overexpression in the UUO- or AD-induced models of renal fibrosis, which was attenuated by SAMiRNA-AREG administration (1 mg/kg or 5 mg/kg). The AREG- and p-EGFR-positive areas were quantified. Scale bar, 100 μm. ***p < 0.001 compared to UUO or AD mice by ANOVA with the Newman-Keuls post-hoc test.

We next determined whether SAMiRNA-AREG regulated EGFR phosphorylation, which is a critical mediator of renal fibrosis and acts as a receptor of AREG. Previous studies confirmed the upregulation of EGFR in renal fibrosis models^28,29^. Moreover, AREG is known to activate EGFR, which amplifies profibrotic downstream signals^17^. IHC staining of AREG showed decreased AREG expression in the SAMiRNA-AREG-treated UUO- or AD-induced mice compared to the UUO or AD mice in which SAMiRNA-AREG was not injected (Fig. 7). We further investigated whether SAMiRNA-AREG influenced EGFR phosphorylation. The IHC staining results showed increased EGFR phosphorylation at Tyr992 in the UUO and AD mouse models, which was reduced by SAMiRNA-AREG treatment (Fig. 7). These results indicated that SAMiRNA-AREG attenuated renal fibrosis by inhibiting AREG and EGFR activation.

### SAMiRNA-AREG downregulates the mRNA expression of fibrosis-related genes induced by TGF-β1 in mouse proximal tubule cells, mouse fibroblasts, and human proximal tubule cells

The differentiation of fibroblasts to myofibroblasts is important in the pathogenesis of fibrotic diseases. TGF-β1 is a key molecule in the induction of myofibroblast differentiation. We performed real-time qRT-PCR to confirm whether SAMiRNA-AREG had potential therapeutic effects in TGF-β1-induced fibrosis. The TGF-β1-stimulated cells showed significant increases in AREG and fibrotic markers, such as α1(I) collagen, fibronectin, and α-SMA (Supplementary Fig. S1-S3). Treatment with SAMiRNA-AREG decreased the mRNA expression of fibrosis markers, as well as AREG, in mouse proximal tubule cells, fibroblasts, and human proximal tubule cells (Supplementary Fig. S1-S3). We confirmed that SAMiRNA-AREG effectively reduced the expression of fibrotic markers induced by TGF-β1.

### AREG is primarily localized in the distal tubules of the UUO and AD model mice

**Figure 8:**
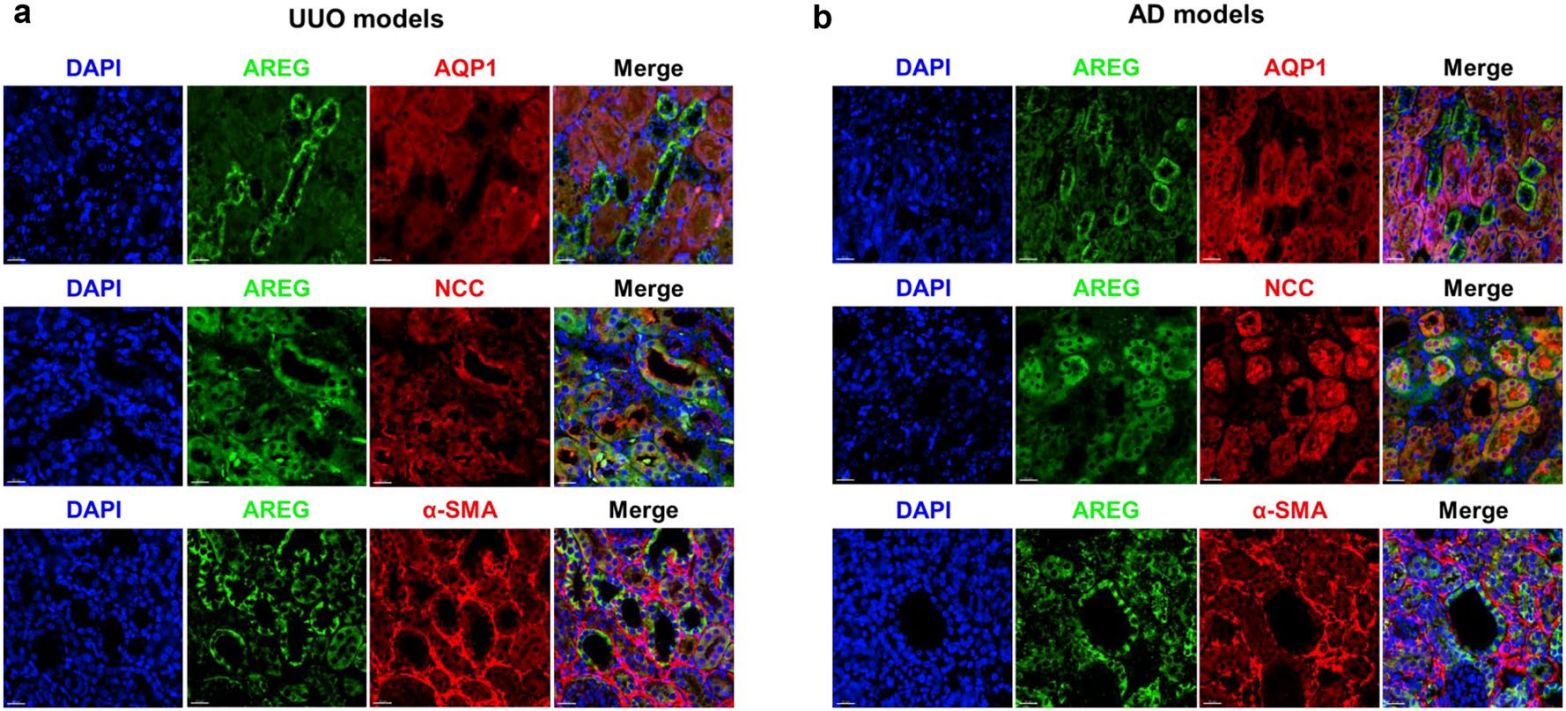
Representative confocal microscopy images of mouse kidney tissues at six days after UUO surgery or seven days after AD. The samples were co-stained for AREG (green), AQP-1 (red, proximal tubule), NCC (red, distal tubule), and α-SMA (red, myofibroblast), and with DAPI to identify the nuclei (blue). Original magnification: × 200. Scale bar, 20 μm.

To clarify the localization of AREG upregulation in the kidneys of the UUO or AD mice, we performed double immunofluorescence staining. Aquaporin-1 (AQP-1), sodium-chloride symporter (NCC), and α-SMA were used as markers of proximal tubules, distal tubules, and myofibroblasts, respectively^30,31^. In both the UUO and AD models, AREG co-localized with NCC, rather than AQP1 or a-SMA (Fig. 8a,b). These results demonstrated that AREG overexpressed in renal fibrosis models was mainly localized in the distal tubules.

## Discussion

In this study, we found that SAMiRNA-AREG exhibited anti-fibrotic effects in the UUO- and AD-induced models of renal fibrosis as well as TGF-β1-induced fibrosis in vitro. The UUO model is a representative animal model of renal fibrosis accompanying tubular interstitial fibrosis^32^. Renal obstruction results in the accumulation of extracellular-matrix (ECM) proteins and increased α-SMA, as well as tubular epithelial cell differentiation into myofibroblasts^32^. In this model, UUO triggers various pathologic changes, such as tubular dilatation, interstitial expansion, hydronephrosis, hypertrophy, and the infiltration of inflammatory cytokines^33^. The accumulation of tubulointerstitial ECM contributes to the progression of fibrosis^34^. Therefore, ECM accumulation in patients with CKD is generally diagnosed based on the symptoms of tubular interstitial fibrosis^33^. Thus, UUO is a good model for studying drug efficacy in CKD and evaluating histopathology. An adenine-rich diet has also been shown to induce renal fibrosis. Adenine is converted to 2,8-dihydroxyadenine, which is accumulated in urine and the renal tubules leading to renal damage via tubulointerstitial nephropathy^27,35^. The AD-induced model of renal fibrosis was recently evaluated using hypoglycemic drugs and fish oil^36,37^. The adenine-induced CKD animal model used in the study reported here was induced by the administration of a 0.2% AD. AREG, an EGFR ligand, is not only associated with lung fibroblast activation, but also plays a regulatory role in fibroblast proliferation and myofibroblast transformation^38^. We confirmed that the systematic injection of SAMiRNA-AREG nanoparticles efficiently silenced target gene expression and significantly improved the pathologic changes associated with dysregulated gene expression. In this study, we employed the target gene *AREG*, which amplifies profibrotic EGFR signals and is a key factor in renal fibrosis^17^.

We found that the inhibition of AREG is an attractive therapeutic strategy in fibrotic disease. Our groups previously demonstrated that SAMiRNA-AREG efficiently knocked down AREG mRNA and protein levels, and fibrotic markers and α-SMA were downregulated by inhibiting AREG in TGF-β transgenic mice and the bleomycin-induced pulmonary fibrosis model^23^.

In UUO models, we performed time-course kinetic experiments in the obstructed renal tissue. AREG expression in the obstructed kidney of UUO mice was significantly higher than in the sham-operated kidneys. Histological analysis showed that SAMiRNA-AREG not only partially decreased the morphological changes in the UUO-induced kidneys but also significantly reduced ECM deposition. Finally, SAMiRNA-AREG suppressed renal injury and fibrosis. In UUO-induced kidneys, F4/80 was significantly increased and the levels of fibrotic markers, as well as inflammatory cytokines, were increased^39,40^. Our groups confirmed that the F4/80 population was decreased after SAMiRNA-AREG treatment in UUO-induced kidneys characterized by upregulated inflammatory cytokines and adhesion molecules via macrophage infiltration^40^. EGFR stimulates fibroblast proliferation and plays a key role in fibrosis^41^. The EGFR signaling pathway is also involved in SMAD2/3-mediated TGF-β1-induced fibrotic conditions^42,43^. AREG/EGFR signaling is a major intracellular signaling pathway in renal fibrosis^17,18^. We found that the AREG increased in UUO-induced kidneys was inhibited by SAMiRNA-AREG treatment. We also confirmed that the upregulated EGFR phosphorylation in UUO-induced kidneys was reversed by SAMiRNA-AREG treatment. Therefore, SAMiRNA-AREG regulates EGFR signaling by inhibiting AREG. These findings suggest that SAMiRNA-AREG nanoparticles represent an effective siRNA modality for intervention in renal fibrosis by downregulating the expression of AREG, fibrosis factors, and inflammatory cytokines.

Figure 5 shows that SAMiRNA-AREG was only accumulated in the UUO kidneys. In general, SAMiRNA is not well-delivered to normal kidney tissue. In contrast, it enters injured or inflamed kidney areas well by the EPR effect. The cell–cell junction of normal epithelial cells on the inner wall of blood vessels is only 2 nm wide. Therefore, SAMiRNA nanoparticles with a size of about 100 ± 20 nm cannot penetrate the junction. In tumors, inflamed, or fibrotic tissue, the cell–cell junction of epithelial cells on the inner wall of blood vessels becomes loose and has leaky vasculature, so nanoparticles with sizes of 50–100 nm can escape into the interstitial space^44^. Fibrosis-specific targeting by SAMiRNA is possible through the passive targeting of the EPR effect, which is common in inflamed or fibrotic tissues^25^. Accordingly, we speculate that the reason SAMiRNA accumulated only in the injured left kidneys was due to the selective delivery of SAMiRNA-AREG to fibrotic tissue (in this case, because of UUO surgery).

We confirmed the time-course experimental findings in AD-induced kidneys. A recent study found that BUN and creatinine were upregulated in the serum of AD-induced mice^36^. We demonstrated that SAMiRNA-AREG improved renal function by reducing serum creatinine and BUN levels. In addition, AD-induced kidneys showed collagen deposits in renal interstitial sites following MT staining^45^. We also evaluated whether the AD-induced collagen accumulation in the kidneys was ameliorated by SAMiRNA-AREG treatment. As shown in the efficacy test results of the UUO model, we found that the expression of fibrotic genes, inflammatory genes, and adhesion molecules in AD-treated kidneys was dramatically increased, but was attenuated by SAMiRNA-AREG. Moreover, we induced fibrotic conditions and experimentally evaluated the efficacy of SAMiRNA-AREG treatment in human and mouse cell lines. After TGF-β1 treatment, the upregulated fibrotic genes were decreased by SAMiRNA-AREG treatment, demonstrating that it was effective even in vitro. This study demonstrated that SAMiRNA-AREG ameliorated kidney injury when administered either subcutaneously or intravenously to mouse models of CKD induced via UUO or AD feeding. Based on the in vitro and in vivo data, we assume that SAMiRNA-AREG attenuated kidney disease in the UUO or AD models and may represent a novel class of RNAi therapeutic drugs for patients with kidney disease.

The novel inhibition pathway of the *AREG* gene using SAMiRNA-AREG had reno-protective effects by ameliorating renal fibrosis and inflammation. Thus, SAMiRNA-AREG is a potential therapeutic siRNA for inhibiting AREG in CKD. Our results demonstrate the safety and efficacy of SAMiRNA-AREG in murine renal fibrosis models.

## Materials and methods

### Animal experiments

All experimental methods and protocols were performed in accordance with the Animal Research: Reporting of In Vivo Experiments (ARRIVE) guidelines and were approved by the Committee on the Ethics of Animal Experiments of the Bioneer Corporation (Daejeon, Republic of Korea, Bioneer-IACUC, AEC-20081229-0004). The Bioneer-IACUC was approved by the Animal and Plant Quarantine Agency (Gimcheon-si, Gyeongsangbuk-do, Republic of Korea). We used 8-week-old male C57BL/6 mice for the UUO kinetic studies. Six mice were included in each group of mice to be examined 0, 2, 4, 6, or 8 days after surgery. To establish the UUO models, we performed surgery to induce UUO as follows. Following anesthesia induction via inhalation of isoflurane, a midline incision was made to expose the kidney and ureter, which was ligated with 6-0 black silk. Sham mice were operated by the same procedure, but without ligating the ureter. Mice that underwent UUO surgery were randomly divided into the day groups described above. The UUO kidneys were harvested on the appropriate days in each group.

The SAMiRNA-AREG efficacy tests in the UUO model mice were performed with 8-week-old male C57BL/6 mice. The mice were divided into four groups: Sham (*n* = 6), UUO with phosphate-buffered saline (PBS) (*n* = 6), UUO with SAMiRNA-AREG 1 mg/kg (*n* = 6), and UUO with SAMiRNA-AREG 5 mg/kg (*n* = 6). SAMiRNA-AREG (1 mg/kg, 5 mg/kg) or PBS was administered to the mice intravenously via the tail vein. The drug was administered after surgery on days 1, 3, and 5. The mice were maintained in individual cages. After 6 days, all the mice were euthanized, and the UUO kidneys were harvested and used for real-time qRT-PCR and histological analysis.

To create the AD models, we used 0.2% AD (TD150071, ENVIGO, Indianapolis, USA). Male 6-week-old C57BL/6 mice were used and six mice were included in each group. Kidneys and blood were harvested at the end of the study. The AD-treated kidneys were used for real-time qRT-PCR, histological analysis, and renal function analyses.

The 6-week-old male C57BL/6 mice used in the SAMiRNA-AREG efficacy tests in the AD model were divided into three groups: control (*n* = 5), AD with PBS (*n* = 5), and AD with SAMiRNA-AREG 5 mg/kg (*n* = 5). The drug was administered subcutaneously after AD on days 0, 1, 2, 3, 4, and 5. The mice were maintained in individual cages. After seven days, all mice were euthanized. The control and AD-treated kidneys were harvested and used for real-time qRT-PCR, histological analysis, and ELISA testing. The serum was analyzed for renal function.

### Ex vivo organ imaging analysis

To confirm whether SAMiRNA-AREG specifically accumulated in the left obstructed kidney, the sham and UUO group were treated intravenously with Cy5-labeled SAMiRNA-AREG at a dose of 5 mg/kg. In the AD renal fibrosis model mice, the Cy5-labeled SAMiRNA-AREG was injected subcutaneously at a dose of 5 mg/kg 1 week after the administration of 0.2% AD. The control group was fed a normal diet for 1 week, and treated with Cy5-labeled SAMiRNA-AREG at a dose of 5 mg/kg subcutaneously. The mice were euthanized 48 h after Cy5-labeled SAMiRNA-AREG administration. Sham, UUO, or AD mice were monitored by in vivo fluorescence imaging and scanned using an IVIS 200 imaging system (Xenogen, Caliper Life Sciences, Hopkinton, MA, USA) under anesthesia with 2.5% isoflurane. Cy5 excitation (λ_ex_ = 640 nm) and emission (λ_em_ = 700 nm) filters were used. The organs of each mouse were collected and scanned. The fluorescence intensity of each kidney was quantified using the Living Image software package (Caliper Life Sciences). The radiant efficiency of the kidney was measured (photons/sec/cm^2^/sr)/(uW/cm^2^), which represents radiance/illumination power density. The background fluorescence was determined before analysis.

### SAMiRNA synthesis and manufacture

SAMiRNA was manufactured as previously described^23^ and briefly included in the Supplementary Materials and Methods. For the injection of SAMiRNA-AREG nanoparticles, we used a solution of 30 mg/ml SAMiRNA-AREG diluted to the desired concentration using PBS (Bioneer, Daejeon, Republic of Korea). The molecular weight of the sense or antisense strand of SAMiRNA-AREG was analyzed using the Axima LNR MALDI-TOF MS system (Kratos Analytical, Shimadzu, UK). The purity of SAMiRNA-AREG was checked by high-performance liquid chromatography (Shimadzu, Kyoto, Japan): sense ≥ 40%, antisense ≥ 40%. The osmolality of SAMiRNA-AREG was measured using Osmomat 3000 (Gonotec, Berlin, Germany) and determined to be 320 ± 30 mOsmol/kg. The size of SAMiRNA-AREG was analyzed with the Zetasizer Nano-ZS (Malvern Panalytical, UK), DynaPro Plate Reader III (Wyatt Technology Corporation, Santa Barbara, CA, USA), or qNano Gold instrument (Izon Science Ltd, Christchurch, New Zealand) and was determined to be ≤ 200 nm.

### Real-time qRT-PCR

The real-time qRT-PCR analysis was performed as previously described^23^. Total RNA was extracted using the AccuPrep Universal RNA Extraction Kit (Bioneer), according to the manufacturer's protocol. The RNA concentration was measured by the absorbance at 260 nm (Tecan, Groedig, Austria). cDNA was prepared from 1 µg of total RNA using the AccuPower RocketScript Cycle RT PreMix (Bioneer) and the AllInOneCycler PCR system. Real-time quantification of the cDNA targets was done using AccuPower 2X GreenStar qPCR MasterMix (Bioneer) and the Exicycler 96 (Bioneer). The mRNA expression of fibrotic markers, inflammatory cytokines, and adhesion molecules was quantitatively analyzed by real-time qRT-PCR. The primer sequences are listed in Supplementary Table S2. RPL13A or GAPDH was used as the housekeeping gene. The data were analyzed via the 2^−ΔΔCt^ method.

### H&E, MT, and IHC staining

The kidney tissues were fixed with 10% neutral-buffered formalin, embedded in paraffin, sectioned at 4 μm, and stained with H&E, as described previously^23^. For the UUO or AD study, the sections were subjected to MT staining to detect collagen fibers following standard procedures. IHC staining was conducted according to the directions of the Novolink Polymer Detection System (Leica Biosystems, Newcastle, UK). Deparaffinized samples were stained with anti-AREG (Bioss, Woburn, MA, USA), anti-α-SMA (Cell Signaling Technology, Danvers, MA, USA), anti-F4/80 (Abcam, Cambridge, UK) or anti-phospho EGFR Tyr992 (Cell Signaling Technology) antibodies. Histological changes were confirmed under a Nikon Eclipse Ts2 microscope (Nikon, Tokyo, Japan). The quantification of tubular dilatation, fibrotic areas, and α-SMA-positive areas was done using ImageJ software (National Institutes of Health, Bethesda, MD, USA).

### Immunofluorescence staining

Paraffin-embedded tissue sections were boiled using a microwave in 10 mM sodium citrate buffer at pH 6.0 for 5 min after deparaffinization. To permeabilize the tissue, the sections were incubated in 0.3% Triton X-100 in PBS for 5 min at room temperature. Nonspecific antibody-binding sites in the tissue sections were blocked with 5% goat serum for 30 min at room temperature. Next, the primary antibodies were added to the tissue sections and the sections were incubated overnight at 4 ℃. To stain the specific markers of renal tubules with AREG, anti-mouse AQP1 (Santa Cruz Biotechnology, Santa Cruz, CA, USA, diluted to 1:200), anti-rabbit NCC (StressMarq Biosciences, Victoria, British Columbia, Canada, diluted to 1:200), anti-mouse AREG (Santa Cruz Biotechnology, dilution ratio 1:100), anti-rabbit AREG (Bioss, Woburn, MA, USA, diluted to 1:100), and anti-α-SMA (Sigma-Aldrich, St. Louis, MO, USA, diluted to 1:200) were used. The next day, fluorescein-conjugated secondary antibodies including anti-rabbit IgG (Alexa Fluor 488 Conjugate, Cell Signaling, Technology, diluted to 1:1,000) or anti-mouse IgG (Alexa Fluor 594 Conjugate, (Cell signaling, diluted to 1:1,000) was added to the tissue sections for 2 h at room temperature. DAPI (4′,6-diamidino-2-phenylindole) was applied to stain the nuclei. Confocal laser scanning images were acquired using a Dragonfly confocal microscopy system (Andor, Belfast, Northern Ireland).

### Cell culture and treatment

Mouse proximal tubule epithelial (mProx24) cells were kindly provided by Kazuo Nakamura (CMIC Co., Ltd, Tokyo, Japan)^46^. NIH-3T3 cells were purchased from the American Type Culture Collection (ATCC, Manassas, VA, USA) and HK-2 cells were obtained from the Korea Cell Line Bank (KCLB, Seoul, Republic of Korea). Cells were seeded in 12-well plates and cultured in media containing 10% bovine calf serum (mProx24) or fetal bovine serum (NIH-3T3 and HK-2), 100 U/mL penicillin, and 100 g/mL streptomycin at 37 °C in a humidified 5% CO_2_ atmosphere, followed by incubation of 70 ~ 80% confluent cells with serum-free media for 24 h and co-treatment with recombinant 10 ng/mL TGF-β1 (R&D Systems, Minneapolis, MN, USA) and SAMiRNA-AREG or PBS for 24 h. The cells were harvested for real-time qRT-PCR analysis.

### Protein sample preparation and ELISA

Kidney tissues were homogenized in RIPA lysis buffer (Thermo Scientific, Pittsburgh, PA, USA). All samples diluted tenfold were quantified using the NanoDrop 2000 (Thermo Scientific). Mouse AREG ELISA kits (R&D Systems) were used according to the manufacturer’s protocol. BUN and creatinine were analyzed using a BUN kit (ARBOR ASSAYS, Ann Arbor, MI, USA) and a creatinine ELISA kit (BioVision, Milpitas, CA, USA), respectively. All ELISA procedures were performed according to the manufacturer’s instructions.

### Statistical analysis

The data are expressed as the mean ± standard error of the mean (SEM). The results were analyzed using a one-way analysis of variance for multiple comparisons between the three groups, followed by the post-hoc Newman-Keuls test. Differences with *p* values of < 0.05 were considered significant.

## Supplementary Information

Supplementary Information.
