# Author Correction: In vivo silencing of amphiregulin by a novel effective Self-Assembled-Micelle inhibitory RNA ameliorates renal fibrosis via inhibition of EGFR signals

**DOI:** 10.1038/s41598-023-38689-3

**Published:** 2023-07-20

**Authors:** Seung Seob Son, Soohyun Hwang, Jun Hong Park, Youngho Ko, Sung‑Il Yun, Ji‑Hye Lee, Beomseok Son, Tae Rim Kim, Han‑Oh Park, Eun Young Lee

**Affiliations:** 1 siRNAgen Therapeutics, Daejeon, 34302 Republic of Korea; 2 Bioneer Corporation, 8‑11 Munpyeongseo‑ro, Daedeok‑gu, Daejeon, 34302 Republic of Korea; 3 Department of Pathology, Soonchunhyang University Cheonan Hospital, Cheonan, 31151 Republic of Korea; 4 Department of Internal Medicine, Soonchunhyang University Cheonan Hospital, 31 Soonchunhyang 6‑gil, Cheonan, 31151 Republic of Korea; 5 Institute of Tissue Regeneration, College of Medicine, Soonchunhyang University, Cheonan, 31151 Republic of Korea; 6 BK21 FOUR Project, College of Medicine, Soonchunhyang University, Cheonan, 31151 Republic of Korea

Correction to: *Scientific Reports* 10.1038/s41598-021-81726-2, published online 26 January 2021

The original version of this Article contained an error in Figure 7, where 'P-EGFR 0 mg/kg' in panel B was a duplication of 'AREG 1 mg/kg' in panel A.

The original Figure 7 and accompanying legend appear below.

**Figure 7:**
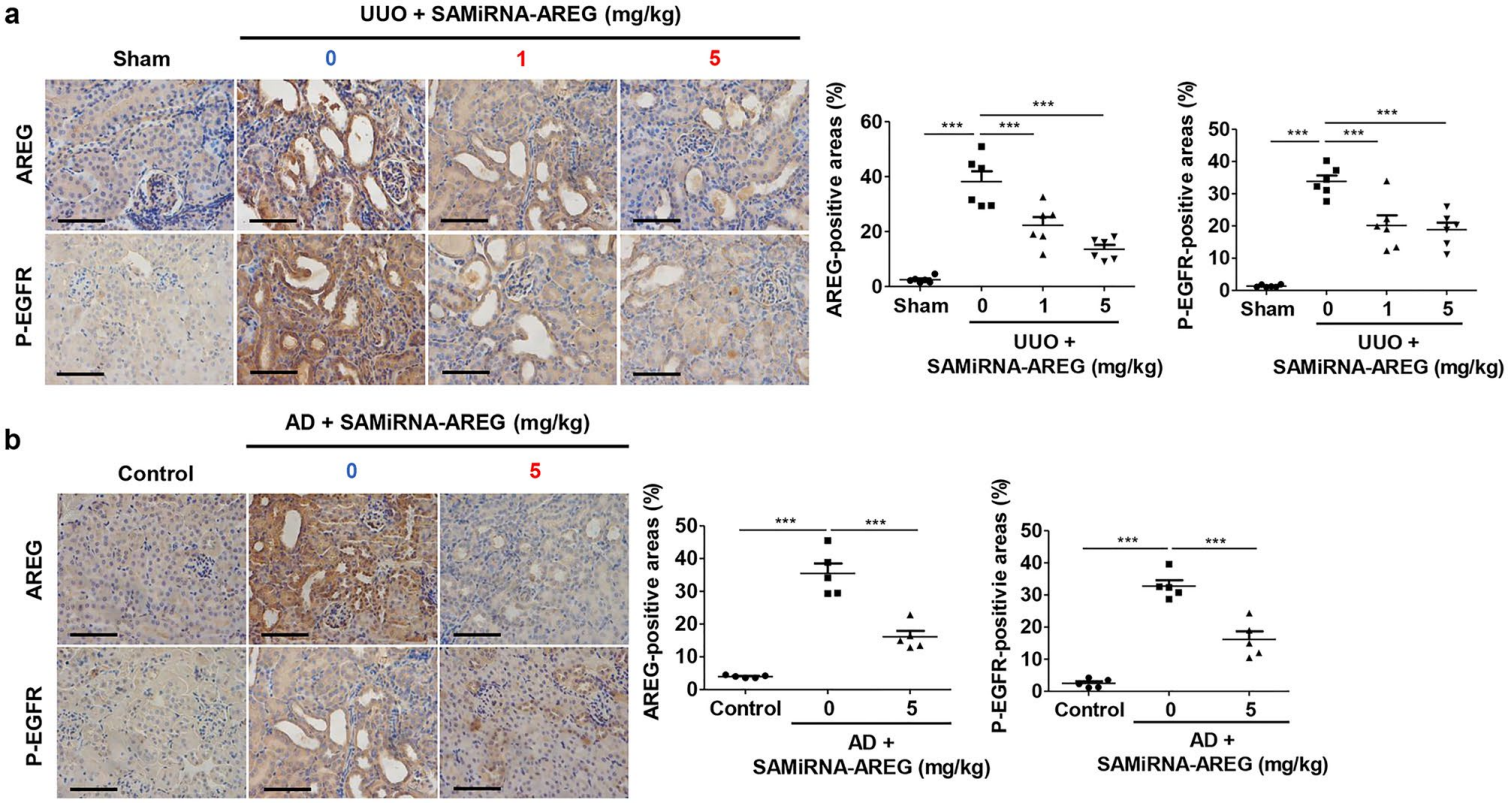
SAMiRNA-AREG inhibited EGFR phosphorylation by downregulating AREG in UUO- or AD-induced renal fibrosis. (**a**,**b**) Representative images of AREG expression and EGFR phosphorylation revealed overexpression in the UUO- or AD-induced models of renal fibrosis, which was attenuated by SAMiRNA-AREG administration (1 mg/kg or 5 mg/kg). The AREG- and p-EGFR-positive areas were quantified. Scale bar, 100 μm. ***p < 0.001 compared to UUO or AD mice by ANOVA with the Newman-Keuls post-hoc test.

The original Article has been corrected.

