# Supplementary material for: In vivo silencing of amphiregulin by a novel effective Self-Assembled-Micelle inhibitory RNA ameliorates renal fibrosis via inhibition of EGFR signals
Source: Sci Rep. 2021 Jan 26;11:2191. doi: 10.1038/s41598-021-81726-2 (PMC7838194; doi:10.1038/s41598-021-81726-2)
Supplement: Supplementary file 1 — Supplementary Information. [file 41598_2021_81726_MOESM1_ESM.pdf]

In vivo silencing of amphiregulin by a novel effective Self-Assembled-Micelle inhibitory RNA ameliorates renal fibrosis via inhibition of EGFR signals

Seung Seob Son[1,7], Soohyun Hwang[1,7], Jun Hong Park[1], Youngho Ko[1], Sung-Il Yun[2], Ji-Hye Lee[3], Beomseok Son[1], Tae Rim Kim[1], Han-Oh Park[1,2*], and Eun Young Lee[4,5,6*]

[1]siRNAgen Therapeutics, Daejeon 34302, Republic of Korea; [2]Bioneer Corporation, Daejeon 34302, Republic of Korea; [3]Department of Pathology, Soonchunhyang University Cheonan Hospital, Cheonan 31151, Republic of Korea; [4]Department of Internal Medicine, Soonchunhyang University Cheonan Hospital, Cheonan 31151, Republic of Korea; [5]Institute of Tissue Regeneration, College of Medicine, Soonchunhyang University, Cheonan 31151, Republic of Korea; [6]BK21 FOUR Project, College of Medicine, Soonchunhyang University, Cheonan 31151, Republic of Korea; [7]These authors contributed equally: Seung Seob Son and Soohyun Hwang.

Correspondence: Eun Young Lee, Department of Internal Medicine, Soonchunhyang University Cheonan Hospital, 31 Soonchunhyang 6-gil, Cheonan 31151, Republic of Korea, Tel: +82-41-570-3684, Fax: +82-41-570-2958, E-mail: eylee@schmc.ac.kr

Han-Oh Park, Bioneer Corporation and siRNAgen Therapeutics, 8-11 Munpyeongseo-ro, Daedeok-gu, Daejeon 34302, Republic of Korea, Tel: +82-42-930-8500, Fax: +82-42-930-8600, E-mail: hpark@bioneer.com

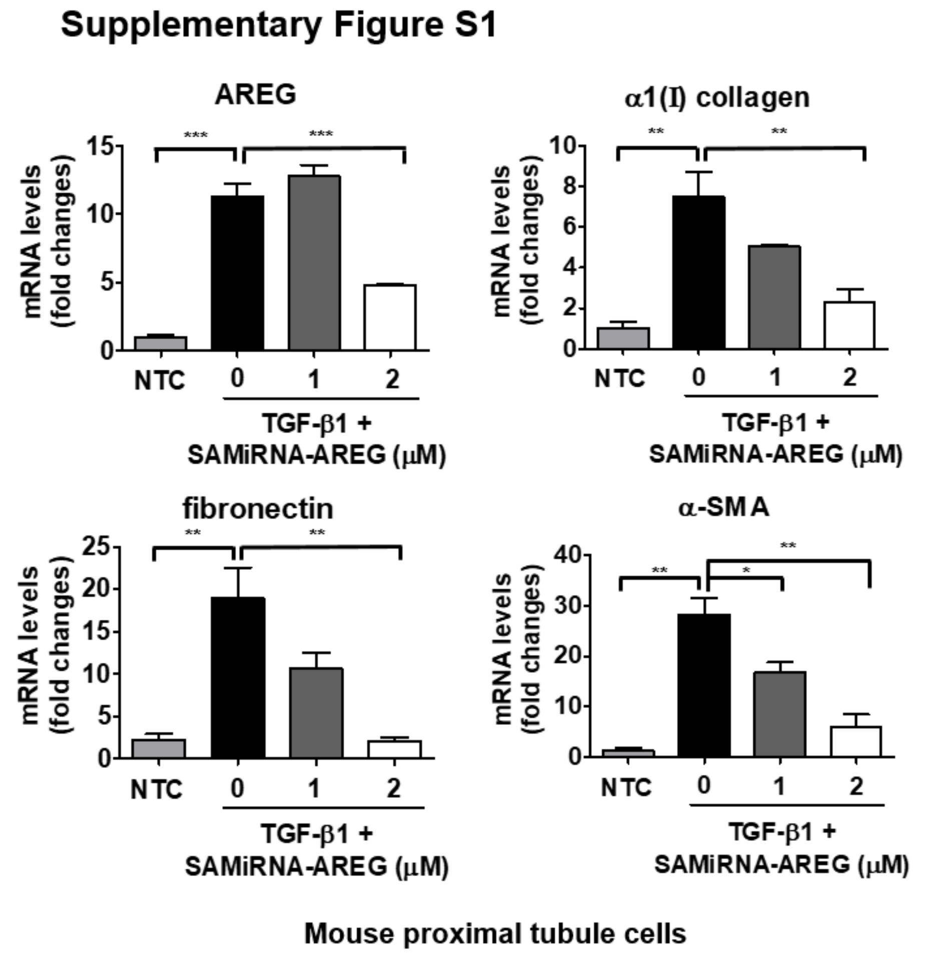

Supplementary Figure S1. SAMiRNA-AREG ameliorated the fibrosis-related mRNA levels during TGF-β1 induction *in vitro*. mProx24 mouse proximal tubule cells were stimulated with or without TGF-β1 (10 ng/mL) for 24 h and co-treated with SAMiRNA-AREG at 1 μM or 2 μM for 24 h. The mRNA expression of AREG, α1(I) collagen, fibronectin, and α-SMA is shown using RPL13A as a reference gene. (mean ± SEM). * $p < 0.05$, ** $p < 0.01$, *** $p < 0.001$ compared to TGF-β1-treated cells by ANOVA with the Newman-Keuls post-hoc test.

Supplementary Figure S2

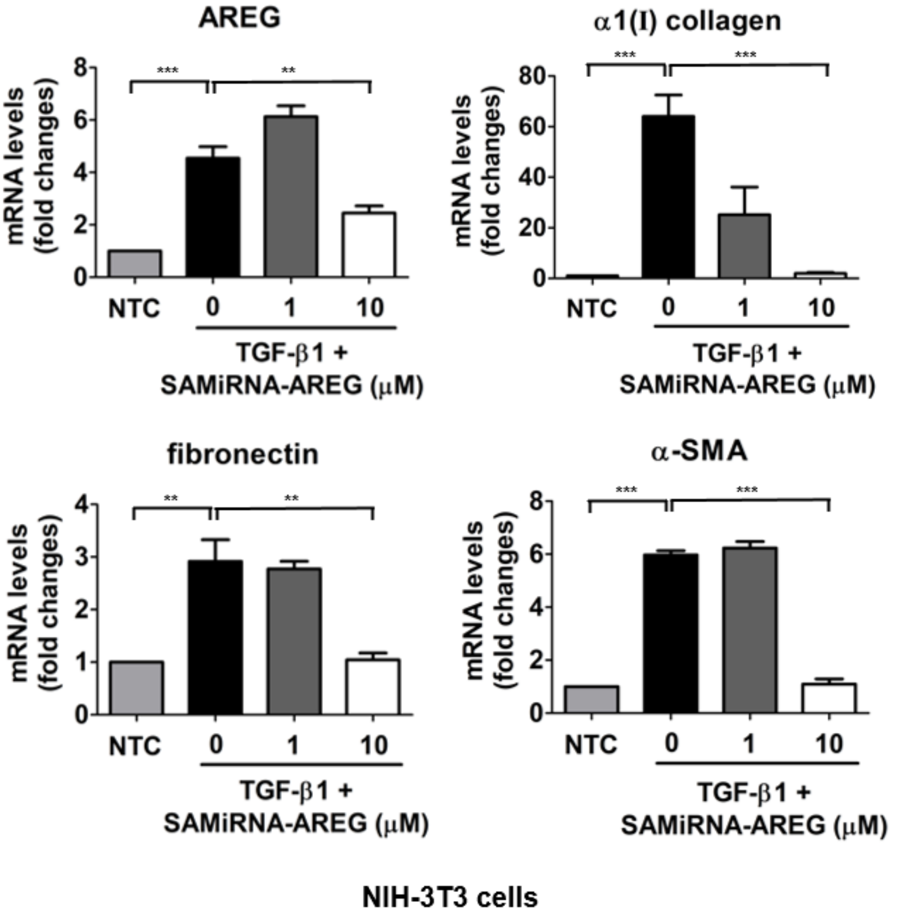

Supplementary Figure S2. SAMiRNA-AREG downregulated fibrosis-related mRNA levels induced by TGF-β1 in NIH-3T3 fibroblasts. NIH-3T3 mouse fibroblasts were stimulated with or without TGF-β1 (10 ng/mL) for 24 h and co-treated with SAMiRNA-AREG at 1 μM or 10 μM for 24 h. The mRNA expression of AREG, α1(I) collagen, fibronectin, and α-SMA is shown using RPL13A as a reference gene. (mean ± SEM). ** $p < 0.01$, *** $p < 0.001$ compared to TGF-β1-treated cells by ANOVA with the Newman-Keuls post-hoc test.

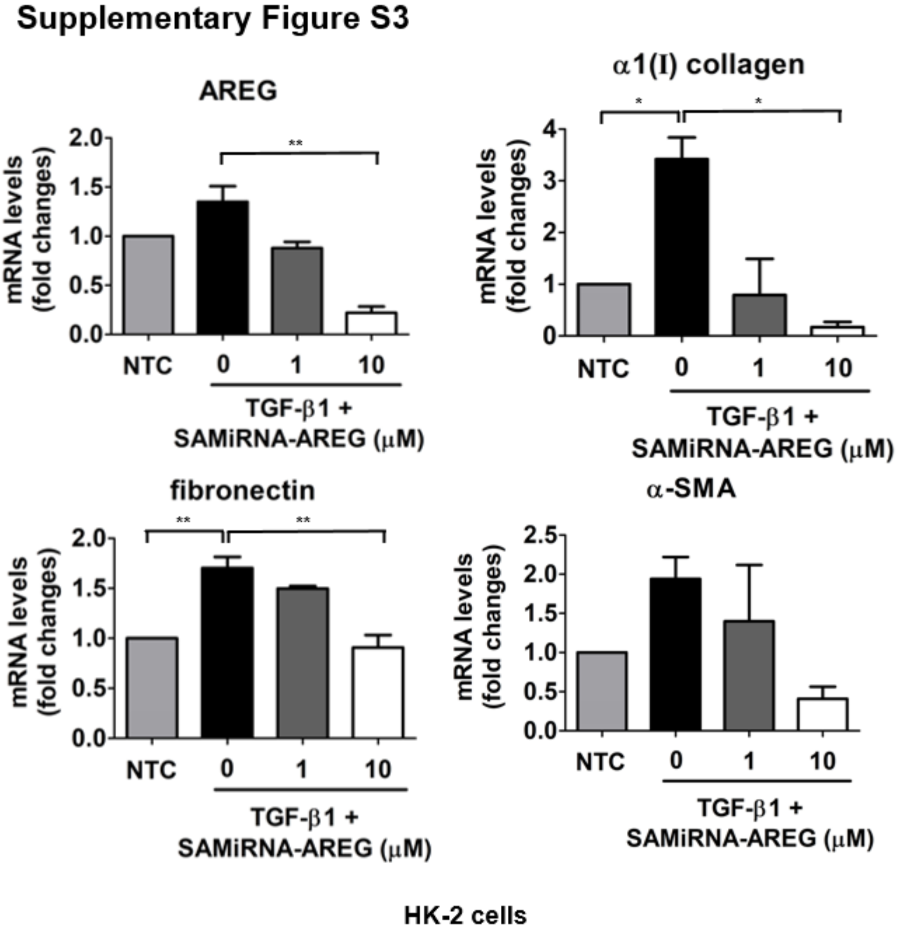

Supplementary Figure S3. SAMiRNA-AREG downregulated fibrosis-related mRNA levels induced by TGF-β1 in HK-2 human proximal tubule cells. HK-2 cells were stimulated with or without TGF-β1 (10 ng/mL) for 24 h and co-treated with SAMiRNA-AREG at 1 μM or 10 μM for 24 h. The mRNA expression of AREG, α1(I) collagen, fibronectin, and α-SMA is shown using GAPDH as a reference gene. (mean ± SEM). * $p < 0.05$, ** $p < 0.01$ compared to TGF-β1-treated cells by ANOVA with the Newman-Keuls post-hoc test.

Supplementary Table S1. Analysis of renal function in UUO- or AD-treated mice. Abbreviations: BW, body weight; BUN, blood urea nitrogen; Cr, serum creatinine. * $p < 0.05$, ** $p < 0.01$, *** $p < 0.001$ compared to controls. Values are expressed as the mean ± SEM.

	**Sham**	**UUO**
BW (g)	24.80 ± 0.79	24.49 ± 0.73
BUN (mg/dl)	21.1 ± 1.07	24.9 ± 1.88
Cr (mg/dl)	0.07 ± 0.01	0.11 ± 0.02

	**Control**	**AD**
BW (g)	22.26 ± 0.02	17.26 ± 0.72***
BUN (mg/dl)	17.95 ± 0.06	89.37 ± 35.08**
Cr (mg/dl)	0.14 ± 0.01	0.48 ± 0.21*

Supplementary Table S2. Sequences of primers used for real-time qRT-PCR

Gene	Species		Sequence
RPL13A	Mouse	Forward	CGATAGTGCATCTTGGCCTTT
		Reverse	CCTGCTGCTCTCAAGGTTGTT
AREG	Mouse	Forward	GAGGCTTCGACAAGAAAACG
		Reverse	ACCAATGTCATTTCCGGTGT
fibronectin	Mouse	Forward	TGGTGGCCACTAAATACGAA
		Reverse	GGAGGGCTAACATTCTCCAG
α-SMA	Mouse	Forward	GGCTCTGGGCTCTGTAAGG
		Reverse	CTCTTGCTCTGGGCTTCATC
α1(I) collagen	Mouse	Forward	TCATCGTGGCTTCTCTGGTC
		Reverse	GACCGTTGAGTCCGTCTTTG
α1(III) collagen	Mouse	Forward	TCACCAGGACAAAGAGGGGA
		Reverse	CCACCAGGACTGCCGTTATT
VCAM-1	Mouse	Forward	ACGAGGCTGGAATTAGCAGA
		Reverse	TTCGGGCACATTTCCACAAG
ICAM-1	Mouse	Forward	GTGCTTTGAGAACTGTGGCA
		Reverse	GGTGAGGTCCTTGCCTACTT
TNF-α	Mouse	Forward	CCTGTAGCCCACGTCGTAG
		Reverse	GGGAGTAGACAAGGTACAACCC
MCP-1	Mouse	Forward	AACTGCATCTGCCCTAAGGT
		Reverse	CTGTCACACTGGTCACTCCT
AREG	Human	Forward	ACACCTACTCTGGGAAGCGT
		Reverse	GCCAGGTATTTGTGGTTCGT
α1(I) collagen	Human	Forward	CCTGGCCCCATTGGTAATGTT
		Reverse	CCCCCTCACGTCCAGATTCAC
fibronectin	Human	Forward	CAAGCCAGATGTCAGAAGC
		Reverse	GGATGGTGCATCAATGGCA

α-SMA	Human	Forward	GATCTGGCACCACTCTTTCTAC
		Reverse	CAGGCAACTCGTAACTCTTCTC
GAPDH	Human	Forward	ATCAAGAAGGTGGTGAAGCAG
		Reverse	GTCGCTGTTGAAGTCAGAGG

Supplementary Materials and Methods

SAMiRNA synthesis and manufacture[23]

1) Preparation of C18-6 Disulfide Phosphoramidite

To bond C18-6 disulfide to a double-helix oligo RNA structure, C18-6 disulfide phosphoramidite was prepared as shown in the following reaction scheme 1.

Reaction scheme 1

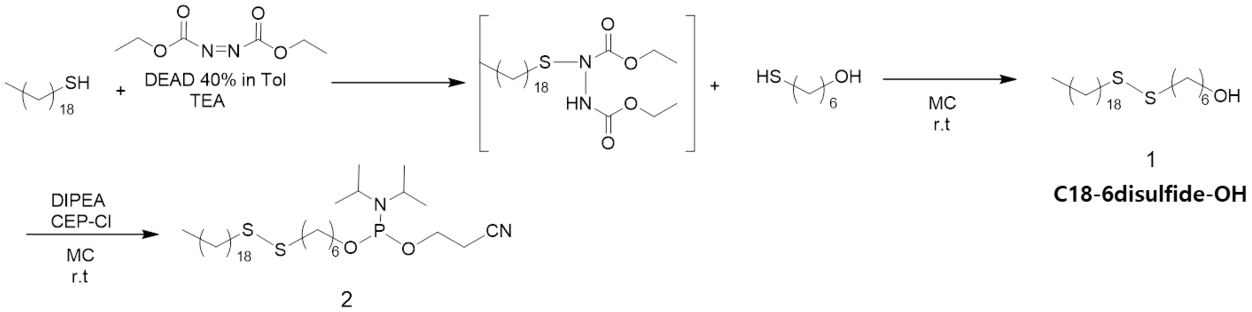

1
C18-6disulfide-OH

2
C18-6 Disulfide Phosphoramidite

2) Preparation of Atom 18 Spacer Phosphoramidite

To bond an Atom 18 Spacer to a double-helix oligo RNA structure, Atom 18 Spacer phosphoramidite was prepared as shown in the following reaction scheme 2.

Reaction scheme 2

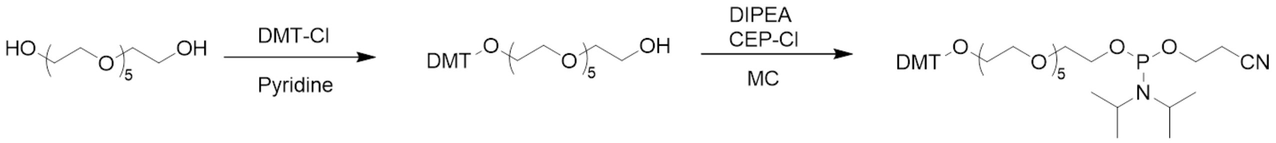

3
DMT-Hexa(ethylene glycol)

4
Atom 18 Spacer phosphoramidite
